# Cellular and Molecular Roles of Human Odorant-Binding Proteins and Related Lipocalins in Olfaction and Neuroinflammation

**DOI:** 10.3390/cells14231859

**Published:** 2025-11-25

**Authors:** Juchan Ha, Hyojin Kim, Hyungsup Kim, Yongwoo Jang

**Affiliations:** 1 Department of Medical and Digital Engineering, College of Engineering, Hanyang University, Seoul 04736, Republic of Korea; hjchan7777@hanyang.ac.kr (J.H.); jine0308@hanyang.ac.kr (H.K.); 2 Research Institute of Pharmaceutical Sciences, College of Pharmacy, Sookmyung Women’s University, Seoul 04310, Republic of Korea; 3 Department of Pharmacology, College of Medicine, Hanyang University, Seoul 04736, Republic of Korea

**Keywords:** olfactory mucus, odorant-binding proteins, lipocalins, chronic rhinosinusitis, neurodegeneration

## Abstract

**Highlights:**

**What are the main findings?**

- Human olfactory mucus proteins, including hOBPs and lipocalins, contribute to odorant transport and mucosal defense, highlighting their broader physiological roles beyond classical chemosensory function.
- The soluble carrier protein repertoire of human olfactory mucus is not dominated by the classical OBPs (OBP2A/2B), which are inconsistently detected, but rather by OBP-like lipocalins (LCN1, LCN2, LCN15, ApoD) and BPI-fold proteins. Together, these proteins coordinate odorant solubilization, antimicrobial defense, redox balance, and ECM remodeling.
- Alterations in these proteins and ECM components are linked to age-related and idiopathic smell loss, potentially involving olfactory signal transduction deficits, and have also been implicated in chronic rhinosinusitis and neurodegenerative disorders.

**What is the implication of the main finding?**

- Emerging evidence highlights the pivotal roles of human olfactory mucus proteins in maintaining sensory signaling, neuronal integrity, and mucosal defense, underscoring the importance of mucus proteomics for understanding olfactory dysfunction and its links to inflammation and neurodegeneration.
- Odorant-binding and carrier proteins functionally interact with ECM components, forming an OBP-like protein–ECM network that sustains olfactory signal transduction and mucosal homeostasis in the human olfactory system.

**Abstract:**

Olfactory perception depends on soluble proteins in the perireceptor environment that support odorant transport, mucosal protection, and tissue homeostasis. In insects, odorant-binding proteins (OBPs) in the sensillum lymph are indispensable for odor detection, whereas in humans the indispensability of OBPs (OBP2A/2B) remains unclear because they are inconsistently detected in nasal mucus. Consequently, it remains unclear whether other soluble proteins compensate for this function or how they contribute to odorant processing and signal transmission within the olfactory mucus. Accumulating evidence indicates that OBP-like lipocalins (LCN1, LCN2, LCN15) and apolipoprotein D, together with bactericidal/permeability-increasing (BPI)-fold proteins, act as major mediators of odorant solubilization, antimicrobial defense, oxidative stress regulation, and extracellular matrix (ECM) remodeling. Alterations in those proteins and ECM organization are linked to idiopathic and age-related smell loss, chronic rhinosinusitis, and neurodegenerative disorders, underscoring their broad relevance at the interface of chemosensation, mucosal defense, and brain health. Major unresolved issues include the functional indispensability of human OBPs, the receptor-specific contributions of OBP-like proteins, and the mechanistic relationships linking olfactory proteome remodeling, sensory signaling, and disease progression. This review provides an integrative overview of structural and mechanistic insights, highlights current controversies, and proposes future research directions, including receptor–protein mapping, integrated structural–functional studies, structural–functional analysis of OBP–ECM networks, and clinical validation of OBP-related biomarkers.

## 1. Introduction

Olfaction is a fundamental sensory modality that enables organisms to detect, discriminate, and respond to chemical cues in the environment. At the molecular level, odorant-binding proteins (OBPs) play pivotal roles in mediating the solubilization, transport, and delivery of hydrophobic odorants to their cognate receptors [1,2,3]. Insects have provided the most compelling experimental evidence for OBP indispensability: the loss of specific OBPs abolishes pheromone recognition and alters behavior [4]. In contrast, mammals appear to rely less directly on OBPs, with odor discrimination driven primarily by the receptor repertoire [2]. Nevertheless, mammalian OBPs, classified as members of the lipocalin family, have been implicated in both odorant transport and semiochemical communication [5,6].

In humans, the genome encodes 10 lipocalins and 9 *LCN*-like genes [7]. Among these, the classical odorant-binding proteins OBP2A (LCN13) and OBP2B (LCN14), together with OBP-like lipocalins such as LCN1, LCN2, LCN15, and apolipoprotein D (ApoD), constitute a diverse group of soluble proteins secreted into the olfactory mucus. In addition to lipocalins, other soluble proteins detected in the human olfactory mucus include members of the BPI (bactericidal/permeability-increasing protein)-fold family, which are primarily involved in innate immune defense. These proteins not only assist in odorant binding and clearance but also participate in mucosal defense, oxidative stress regulation, and extracellular matrix (ECM) remodeling [1,2]. Importantly, recent proteomic studies suggest that alterations in these proteins are associated with olfactory dysfunction, including idiopathic and age-related smell loss—where LCN15, fibronectin, and HSP70 show reduced expression—and various pathological conditions, including chronic rhinosinusitis, in which LCN2 and BPIF proteins are notably dysregulated.

Despite those results, substantial controversies remain, most notably concerning the essentiality of human OBPs and the reliability of their detection in proteomic analyses. In particular, the consistent detection of classical OBPs in nasal mucus has proved challenging, fueling debate about their indispensability in human olfaction [2]. Moreover, the extent to which OBP-like proteins act as broad carriers versus receptor-specific modulators remains unclear. Furthermore, emerging evidence linking alterations in the olfactory proteome to neuroinflammatory and neurodegenerative processes highlights the significance of OBPs beyond classical chemosensory biology.

For this review, we conducted a comprehensive literature search using PubMed, Web of Science, Scopus, and Google Scholar. Boolean combinations of keywords were employed, including “odorant-binding protein,” “lipocalin,” “olfactory mucus,” “nasal mucus proteome,” “olfactory cleft,” “BPI-fold,” and “PLUNC,” together with terms such as “olfactory dysfunction,” “chronic rhinosinusitis,” “extracellular matrix,” and “neurodegeneration.” Priority was given to studies presenting original experimental data on human olfactory or nasal mucus proteins, particularly those involving mass spectrometry–based proteomic profiling, functional biochemical analyses, immunohistochemical localization, and disease association studies. Comparative findings from mammalian model systems and insect olfactory systems were also selectively incorporated when they provided mechanistic insights or evolutionary perspectives relevant to human olfactory biology. This review provides an integrated framework of the human olfactory mucus proteome by combining structural, functional, and disease-oriented perspectives. It emphasizes the molecular interplay between soluble carrier proteins and ECM components as a key determinant of olfactory signaling and tissue homeostasis. By examining the structural and functional diversity of mammalian OBPs alongside the cooperative roles of binding proteins and ECM molecules within the human olfactory mucus, this review elucidates how these interactions contribute to sensory signaling, mucosal defense, and homeostatic regulation. It further highlights the physiological relevance, existing knowledge gaps, and potential diagnostic and therapeutic implications of mucus-associated binding proteins in olfactory dysfunction, chronic inflammation, and neurodegenerative diseases.

## 2. Structural and Functional Diversity of Odorant-Binding Proteins

### 2.1. Discovery and Evolutionary Divergence of OBPs

Odorant-binding proteins (OBPs) are soluble, secreted carriers with hydrophobic cavities that enable the solubilization and transport of odorants. These proteins were first identified contemporaneously nearly four decades ago in the nasal mucus of cattle and the antennae of the giant moth *Antheraea polyphemus* [4]. Since those initial discoveries, DNA sequences encoding OBPs have been identified across a wide range of species, from insects to mammals. Insects possess OBPs in more than one hundred species, including *Drosophila melanogaster* and the silkworm *Bombyx mori* [8,9], while vertebrate OBPs have been characterized in rodents, pigs, and humans [10,11]. Comparative studies have further demonstrated that the functions and structural features of OBPs diverge substantially between insects and mammals in ways that reflect distinct evolutionary adaptations. Insects rely extensively on OBPs to detect and transport a broad spectrum of environmental chemical cues. Within their olfactory and gustatory sensilla, the dendrites of sensory neurons are bathed in sensillar lymph, where both volatile and non-volatile compounds are solubilized and subsequently shuttled to their cognate receptors through perireceptor events. Perireceptor events refer to a series of extracellular processes that occur between odorant entry into the mucus and receptor activation. These include the solubilization of hydrophobic odorants by carrier proteins such as OBPs and lipocalins, their guided diffusion or transport through the mucus, potential enzymatic modification or degradation of odorants, and eventual clearance after receptor binding. Collectively, these coordinated processes regulate odorant availability, concentration dynamics, and temporal resolution, thereby shaping the initial stage of olfactory signal transduction and ensuring precise receptor activation [3].

Foundational studies on insect OBPs provided the conceptual and methodological groundwork for understanding odorant transport mechanisms, which later guided investigations into mammalian counterparts. Early discoveries in insects, such as ligand binding, pH-dependent release, and structural stabilization, established core principles that inspired analogous hypotheses in vertebrate systems. Although mammalian OBPs are structurally and evolutionarily distinct, their comparable biochemical strategies for odorant solubilization and delivery represent an example of functional convergence rather than direct evolutionary continuity in odorant transport systems [10,12,13,14].

Despite this functional analogy, insect and mammalian olfactory systems differ markedly in their receptor architectures and signal transduction pathways. Insects use non-canonical ligand-gated ion channels that respond directly to odorant molecules and enable rapid ion transmission, whereas mammals depend on GPCR-based receptors within the nasal mucus layer [15]. This GPCR-coupled signaling cascade in mammals enables more flexible modulation and fine-tuning of sensory responses through second messenger amplification and multiple regulatory checkpoints, allowing for greater adaptation to varying odorant concentrations and environmental contexts. In addition, individual insect olfactory sensory neurons can co-express multiple receptors, thereby broadening their tuning ranges and adding flexibility to their odor detection capabilities. In contrast, mammalian olfactory neurons generally adhere to the ‘one receptor–one neuron’ rule, whereby each neuron expresses only one functional odorant receptor gene from approximately 400 intact receptor genes in the human genome [16]. Nonetheless, the olfactory epithelium contains millions of neurons, with many thousands expressing the same receptor type and converging onto specific glomeruli in the olfactory bulb [16], thereby ensuring receptor-specific signaling and amplification. Beyond those neuronal and receptor-level differences, a distinction emerges at the level of OBPs. In insects, OBPs mediate the solubilization and transport of environmental odorants to receptors within the sensillar lymph. They also play an important role in pheromone detection, as exemplified in *Drosophila*, where the OBP LUSH is indispensable to social chemical communication [17,18]. In mammals, OBPs that diverge structurally from their insect counterparts have been proposed to serve multiple roles in chemical communication. Insect OBPs are characterized by a compact six-alpha-helix bundle stabilized by three conserved disulfide bonds, typically comprising 120–150 amino acids. In contrast, mammalian OBPs belong to the lipocalin superfamily and feature an eight-stranded antiparallel β-barrel that forms a calyx-shaped binding pocket capped by an α-helical domain. This fundamental architectural difference reflects independent evolutionary origins and convergent evolution for analogous odorant-binding functions. Moreover, insect OBPs exhibit extreme sequence diversity even among closely related species and often regulate ligand binding and release through pH-dependent conformational changes, whereas mammalian OBPs show greater structural conservation across species and use different mechanisms such as quaternary structural variation and post-translational modification for ligand interaction. One suggested role of mammalian OBPs is the solubilization and transport of volatile pheromones through the nasal mucus to olfactory receptors [2]. In addition, mammalian OBPs can be detected in various biological fluids, including urine, saliva, and sexual secretions, where they might contribute to species-specific pheromonal signaling by transporting odorants. A notable exception is the family of major urinary proteins (MUPs) in rodents, which share the same β-barrel carrier architecture and transport volatile compounds, yet some members can also act directly as pheromonal signals themselves [19]. However, unlike in insects, their indispensability for odor detection remains unresolved.

### 2.2. Structural Features of OBPs Across Species

**Figure 1:**
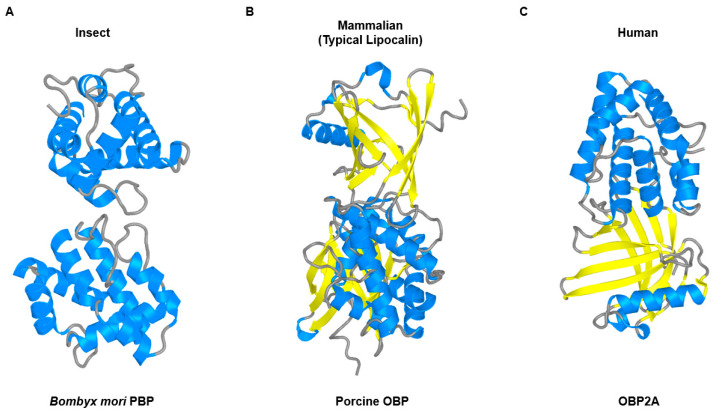
Structural comparison of odorant-binding proteins (OBPs) across different species. Three representative OBP structures highlighting the distinct architectural solutions for odorant transport. (**A**) Insect OBP (*Bombyx mori* PBP, PDB: 1DQE) shown as a functional dimer displaying the six α-helical secondary structures, and gray shows loop regions. The compact fold is stabilized by three conserved disulfide bridges (Cys19-Cys54, Cys50-Cys108, Cys97-Cys117); (**B**) Mammalian OBP (Porcine OBP, PDB: 1A3Y) shown as a monomer exhibiting the classical lipocalin fold featuring an eight-stranded antiparallel β-barrel. Yellow ribbons depict the β-sheets forming the barrel structure, while blue represents α-helices including the C-terminal helix that caps the barrel. Gray shows loop regions connecting the secondary structure elements. (**C**) Human OBP2A (PDB: 4RUN) shown as a monomer displaying the characteristic lipocalin architecture with an eight-stranded β-barrel. Yellow ribbons represent the β-sheet barrel, bluehighlights the α-helical elements and important structural features including the reactive cysteine (Cys59) at the binding site. This is an original figure created by the authors using structural data from the indicated PDB entries, visualized with PyMOL3.1.

Despite their similar functional roles, OBPs display distinct structural characteristics across species, reflecting divergent evolutionary adaptations for odorant transport as depicted in Figure 1. These structural differences represent not simple species-level divergence, but distinct molecular strategies optimized for the specific physicochemical environments in which these proteins function.

Insects use compact OBPs composed of six α-helices that are stabilized by three conserved disulfide bridges to form a hydrophobic cavity optimized for binding small volatile ligands [9,20]. This characteristic six-helix fold represents a remarkably robust structural motif that is preserved across diverse insects, despite extreme sequence divergence. Insect OBPs often share less than 10% sequence identity even within a single species, reflecting rapid evolutionary diversification unique to insect OBP subfamilies, a pattern not observed in mammalian lipocalins [21].

Insect OBPs have diversified into several types and are classified by their cysteine patterns. In Diptera, they are divided into five distinct subfamilies based on the number and arrangement of conserved cysteines: (1) classic OBPs with the typical six-cysteine signature, (2) dimer OBPs containing two six-cysteine signatures, (3) plus-C OBPs with two additional conserved cysteines plus one proline, (4) minus-C OBPs that have lost two conserved cysteines, and (5) atypical OBPs with 9–10 cysteines and an extended C-terminus [22,23,24]. More than 20 high-resolution crystal and nuclear magnetic resonance structures consistently demonstrate this conserved fold in both apo- and ligand-bound states, underscoring the structural robustness of the OBP scaffold across diverse binding contexts [25,26]. Within this conserved structural framework, insects have evolved specialized functions adapted to their ecological niches. OBP1 from *Anopheles gambiae* and *Aedes aegypti* illustrates one such adaptation, a dimeric architecture that forms a continuous tunnel spanning both subunits. This configuration provides a distinctive binding topology not observed in monomeric forms [27,28]. Lepidopteran pheromone-binding proteins exemplify another sophisticated mechanism: pH-dependent conformational switches in their C-terminal domains regulate ligand release, thereby ensuring precise odorant delivery at target membranes [29,30]. Those findings highlight a paradoxical property of insect OBPs: they are characterized by extreme sequence variability coupled with strong structural conservation. This unique combination allows functional diversification in odorant delivery kinetics and binding selectivity, which can modulate the efficiency and temporal dynamics of receptor activation, while preserving a stable six-helix core optimized for the solubilization, transport, and regulated release of hydrophobic odorants [31]. However, odor perception and discrimination are ultimately determined by olfactory receptor diversity and neuronal coding, rather than by OBP specificity. It has given rise to a highly versatile protein family that balances structural stability with functional diversity, enabling insects to adapt their chemosensory systems to diverse ecological challenges.

Whereas insect OBPs are built on a compact six-helix fold stabilized by disulfide bridges, mammalian OBPs are members of the lipocalin family, a large group of secreted proteins characterized by an eight-stranded antiparallel β-barrel capped by a C-terminal α-helix. This structural framework also forms a central hydrophobic pocket optimized for binding small volatile molecules [32]. The conserved lipocalin fold is not unique to mammalian OBPs but is also shared by related proteins such as major urinary proteins, salivary lipocalins, and von Ebner’s gland proteins, all of which contribute to semiochemical communication and odorant transport [2]. Despite the conserved fold, mammalian OBPs exhibit notable structural plasticity in their quaternary organization. Most OBPs exist as monomers, but the bovine OBP adopts a homodimeric configuration through domain swapping, thereby generating novel binding interfaces beyond the canonical pocket [33]. In contrast, the porcine OBP, crystallized in its monomeric state, retains the canonical lipocalin barrel with cavity features that support the broad recognition of odorants and pheromones [34]. In humans, two OBP genes have been identified, designated OBP2A and OBP2B. OBP2A exhibits broad tissue expression, including the nasal mucosa, lungs, salivary glands, lacrimal glands, and reproductive tissues, whereas OBP2B expression is largely restricted to the prostate and mammary glands [35]. Despite those distinct expression profiles, the two proteins share approximately 90% sequence identity, suggesting nearly identical structural and functional properties [35]. The crystal structure of human OBP2A confirms the conserved lipocalin β-barrel fold, which is characterized by a large hydrophobic binding pocket, positively charged loops at the cavity entrance, and a reactive cysteine located within the binding site [35]. However, proteomic analyses of human olfactory mucus have yielded inconsistent evidence for the presence of OBP2A/2B, raising ongoing questions about their precise contribution to human olfaction, an issue that will be addressed in more detail in Section 3.

Therefore, the contrasting architectures of OBPs across species represent alternative structural solutions to a common physiological challenge in which these proteins solubilize and transport hydrophobic odorants within extracellular environments that differ fundamentally in their physical and chemical properties.

### 2.3. Mechanistic Comparison of Odorant Binding

The mechanisms by which odorants interact with OBPs are diverse and closely linked to the underlying protein fold. In insects, three characteristic modes of odorant binding have been identified. First, odorant binding can rely on quaternary structural arrangements. *Anopheles gambiae* OBP1 and *Aedes aegypti* OBP1 form dimers with a continuous inter-subunit tunnel that accommodates odorants, and those bound ligands are subsequently released through pH-triggered gating of the trans-dimer channel [36,37]. Such bound odorants can also be subject to a second mode of regulation involving pH-dependent conformational switches. In *Bombyx mori* pheromone-binding protein 1 (PBP1), acidification induces a coil-to-helix transition in the C-terminal segment that inserts into the binding pocket and promotes ligand release near the receptor surface [25]. A third mechanism is conformational transduction. Upon binding the male pheromone cis-vaccenyl acetate (cVA), *Drosophila* LUSH undergoes a ligand-specific conformational change that is required for the activation of OR67d-expressing neurons [38,39,40].

Mammalian OBPs use distinct mechanisms centered on the β-barrel fold. In these proteins, variation in ligand interaction arises primarily from quaternary organization and chemical modifications, rather than pH-driven gating. For example, bovine OBP adopts a domain-swapped dimeric architecture that generates an interfacial binding site in addition to the canonical cavities, thereby broadening ligand accommodation [41,42]. Post-translational modifications provide another layer of functional diversity; for example, porcine OBP isoforms generated by O-GlcNAcylation and phosphorylation exhibit distinct odorant specificities [43,44]. Human OBPs generally follow the mammalian paradigm and do not use pH-dependent mechanisms. OBP2A specificity is largely determined by the geometry of the β-barrel and key side chains at the cavity entrance, particularly Lys112 for hydrogen bonding and Phe97 for π–π interactions [1,35]. This binding mechanism is relatively simple compared with the dynamic conformational changes that characterize many insect OBPs. Taken together, these comparisons highlight the evolutionary divergence of binding mechanisms between insects and mammals.

This divergence ultimately reflects the distinct extracellular environments in which OBPs function. While insect OBPs operate within the confined, hydrophobic sensillar lymph—conditions that favor compact α-helical scaffolds stabilized by disulfide bridges and capable of pH-triggered conformational switching—mammalian OBPs have evolved under markedly different biochemical constraints. In the aqueous, protein-rich nasal mucus of the olfactory cleft, chemical complexity, oxidative stress, and abundant ECM components demand greater structural stability and ligand versatility [45]. In this environment, lipocalin-type OBPs interact with ECM proteins and other mucus constituents to form a dynamic perireceptor network that modulates odorant transport, mucosal protection, and sensory resilience [46]. These cooperative interactions collectively define the proteomic landscape of the human olfactory cleft, where OBPs, lipocalins, apolipoproteins, and ECM molecules integrate to regulate odorant processing and epithelial homeostasis, as discussed in the following section [2].

## 3. Proteomic Landscape of the Human Olfactory Cleft

### 3.1. Odorant-Binding Proteins

The human olfactory cleft contains a complex proteomic environment comprising proteins that participate in odorant detection, metabolic processing, and mucosal defense as summarized in Table 1. The proteins included in Table 1 were selected based on three primary criteria: (1) consistent detection across multiple independent proteomic studies of human olfactory or nasal mucus, indicating biological relevance and abundance; (2) experimentally demonstrated or strongly proposed functional roles in odorant binding, transport, metabolism, mucosal defense, or tissue homeostasis that may influence olfactory signaling; and (3) availability of structural or biochemical characterization providing mechanistic insights. This functionally curated subset emphasizes proteins with established relevance to olfactory physiology rather than representing a comprehensive catalog of all detectable proteins in the olfactory cleft. In 2000, Lacazette et al. reported that OBP2A was expressed in the nasal mucosa and associated secretory glands, whereas OBP2B showed little to no expression in the nasal mucosa but was prominently expressed in reproductive tissues, including the prostate and mammary glands. Both OBP2A and OBP2B exhibit alternative splicing, resulting in isoforms with unique C-terminal regions [47]. Subsequently, proteomic analyses detected OBP2A in human olfactory mucus [10,48]. Those studies demonstrated that OBP2A binds a broad spectrum of hydrophobic odorants, with particularly high affinity for aldehydes and long-chain fatty acids. Later, Tcatchoff et al. identified Lys112 as a critical residue mediating the enhanced affinity of OBP2A for aldehydes, thereby providing a structural basis for its binding specificity [49]. Although the physiological role of OBP2A in human olfactory mucus has not yet been fully defined, it has been proposed to function not only as an odorant carrier but also as a protective factor against toxic hydrophobic compounds. OBP2A has been shown to be stably expressed and secreted in keratinocytes and reconstructed 3D epidermal models, where it captures lipid peroxidation products and environmental toxins to support barrier integrity and mitigate oxidative stress. Those findings suggest that OBP2A might also play a protective role in nasal mucus [50]. In contrast, the presence of OBP2B in human nasal mucus has not been confirmed at either the transcript or protein level. More recent evidence, however, indicates that a regulatory enhancer linked to the ABO blood group can modulate OBP2B expression [51]. Despite those observations, large-scale untargeted proteomic analyses have failed to consistently detect OBP2A or OBP2B in olfactory cleft mucus [52,53]. Earlier proteomic studies using 2-DE/MALDI-TOF identified approximately 80 proteins but similarly failed to reliably capture OBPs [48].

### 3.2. OBP-like Lipocalins

In the absence of consistently detectable classical OBPs, other lipocalins are abundantly present in human olfactory mucus. While these proteins possess hydrophobic binding pockets characteristic of the lipocalin fold and can bind lipophilic molecules, their primary established functions relate to mucosal defense, oxidative stress regulation, and tissue homeostasis rather than direct odorant transport to receptors. Lipocalins are a family of evolutionarily conserved, low-molecular-weight (18–40 kDa) proteins widely distributed across taxa. The human genome encodes 10 lipocalins (*LCN1*, *LCN2*, *LCN6*, *LCN8*, *LCN9*, *LCN10*, *LCN12*, *LCN13* [*OBP2A*], *LCN14* [*OBP2B*], and *LCN15*) and 9 *LCN*-like genes (*AMBP*, *APOD*, *APOM*, *C8G*, *ORM1*, *ORM2*, *PAEP*, *PTGDS*, and *RBP4*)—a smaller repertoire than that found in the mouse genome [7]. These lipocalins exhibit distinct tissue distributions and specialized physiological roles. Within the olfactory system, LCN1, LCN2, LCN15, and APOD are the most consistently detected members, contributing to mucosal defense, oxidative balance, and odorant signaling. Other members, including LCN8, LCN9, LCN12, and LCN13, are primarily expressed in the epididymis and are associated with sperm maturation and lipid or retinoid transport [67,68]. More recently, LCN10 has been implicated in systemic immune and vascular regulation [69], although its role in the olfactory system remains to be elucidated. LCN1 is secreted from accessory glands of the nasal and oral cavities and binds a broad spectrum of hydrophobic ligands, including fatty acids, fatty alcohols, phospholipids, glycolipids, and cholesterol [70]. LCN1 has also been shown to transport antimicrobial fatty acids such as lauric acid, thereby contributing to bacterial growth inhibition [71,72]. In addition, it can scavenge lipid peroxidation products within the mucus layer, suggesting a protective role against oxidative stress [73,74]. The distribution of LCN1 is not limited to the olfactory region but extends throughout the nasal cavity, where it might contribute to general mucosal defense and the maintenance of olfactory function [74]. Moreover, LCN1 binds bacterial catecholate siderophores such as enterobactin, hydroxamate siderophores such as desferrioxamine B, and even major fungal siderophores, thereby positioning it as a novel innate immune factor in mucosal defense [75].

Structural studies have revealed a dual binding mode for fatty acids within the LCN1 cavity, a feature that might stabilize the mucus lipid layer and help preserve the olfactory signaling environment [76]. In addition, LCN1 has been shown to interact with phospholipid transfer protein to mediate phospholipid exchange, a mechanism that contributes to the organization and stability of tear film [56]. Taken together, this evidence suggests that LCN1 performs similar functions in the olfactory mucus, contributing to lipid homeostasis and strengthening mucosal protection. On the other hand, LCN2 (also known as NGAL) is secreted into nasal mucus by epithelial and immune cells, where it chelates bacterial siderophores and restricts microbial access to iron, thereby contributing to antimicrobial defense [55,77]. Its presence has also been confirmed in nasal tissues, supporting a potential role in maintaining iron homeostasis and mucosal integrity. LCN15 is a human-specific member of the lipocalin family that is selectively enriched in olfactory cleft mucus, where it is produced and secreted by Bowman’s glands [54]. Notably, LCN15 immunoreactivity correlates with regions containing non-degenerated olfactory sensory neurons, suggesting that neuronal preservation is closely associated with mucus secretion and LCN15 abundance [54]. A recent study further suggests that LCN15 is more than a structural mucus component: it has been shown to enhance odorant signaling for specific receptor–odorant pairs, such as OR51E1–isovaleric acid [78]. Those findings suggest that LCN15 can augment odorant responses in a receptor-specific manner, although its broader contribution across the human olfactory receptor repertoire remains to be elucidated. ApoD is a glycoprotein with a characteristic lipocalin fold that has been consistently identified in proteomic analyses of human olfactory mucus and has been described as an OBP [52,53,79]. In human axillary apocrine secretions, the odorant precursor E-3-methyl-2-hexenoic acid was found to associate with two OBPs (ASOB1 and ASOB2), and ASOB2 was identified as ApoD [80]. Supporting that role, Zhu et al. (2015) reported remarkably elevated ApoD mRNA levels in patients with osmidrosis, indicating that ApoD is expressed in apocrine glands and contributes to odor precursor transport [81]. Although ApoD exhibits odorant-binding capacity, particularly in axillary secretions, its role in odor perception within the olfactory cleft remains uncertain. The detection of ApoD and other lipocalins in olfactory mucus therefore indicates potential supportive functions in maintaining mucosal integrity, rather than direct substitution for classical OBPs in odorant delivery. Beyond its odorant-related functions, ApoD has also been implicated as a protective protein in the context of oxidative stress and inflammation [82,83,84]. Mechanistically, ApoD binds arachidonic acid released from membrane phospholipids and lipid hydroperoxides generated either endogenously during oxidative stress or introduced from the external environment, thereby limiting both inflammatory signaling and toxic insults.

### 3.3. BPI-Fold/PLUNC Family

**Figure 2:**
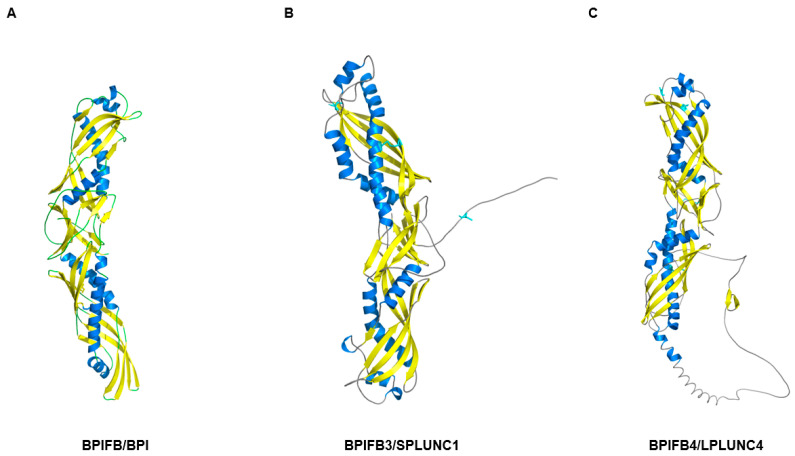
Structural comparison of human BPIFB subfamily proteins. Three representative structures demonstrating the conserved two-domain BPI-fold architecture within the BPIFB (long PLUNC) subfamily. (**A**) BPIFB/BPI (PDB: 1BP1) represents the archetypal two-domain BPI-fold protein exhibiting the characteristic extended boomerang-shaped structure. Blue ribbons depict alpha-helical secondary structure elements, yellow highlights beta-strand regions forming the central core, and gray shows flexible loop regions connecting the structural elements. The two tandem BPI-fold domains create spatially separated hydrophobic pockets on the concave surface that mediate lipid binding and antimicrobial activity. (**B**) BPIFB3/SPLUNC1 (AlphaFold3 prediction, model_0, pTM = 0.79) maintains the conserved boomerang-shaped architecture despite sharing only approximately 25% sequence identity with BPI. The predicted structure exhibits high confidence across most regions (pLDDT > 90), with the N-terminal and C-terminal regions showing slightly lower confidence values typical of terminal flexibility. The overall fold topology remains consistent with the canonical BPI-fold, validating structural conservation within the family. (**C**) BPIFB4/LPLUNC4 (AlphaFold3 prediction, model_0, pTM = 0.67) represents another member of the long PLUNC subfamily expressed predominantly in olfactory epithelium. The AlphaFold3 prediction reveals a two-domain structure with moderate overall confidence (pTM = 0.67), reflecting the challenges in predicting interdomain orientations and flexible linker regions typical of multi-domain proteins. The confidence map shows predominantly high pLDDT values (>90) in the structured core regions of both N-terminal and C-terminal domains, with lower confidence in the extended C-terminal tail and interdomain linker regions, consistent with intrinsic flexibility in these segments. The two BPI-fold domains maintain the characteristic secondary structure composition and fold topology despite sequence divergence, demonstrating robust conservation of the architectural framework across the BPIFB subfamily. This is an original figure created by the authors using structural data from the indicated PDB entry and AlphaFold3 predictions, visualized with PyMOL3.1.

BPI (bactericidal/permeability-increasing protein), LBP (LPS-binding protein), and PLUNC (palate, lung, and nasal epithelium clone) family proteins share a conserved structural motif known as the BPI-fold. This fold generates a boomerang-shaped hydrophobic pocket that facilitates the recognition and binding of lipids, such as bacterial lipopolysaccharides (LPS) and fatty acids [85]. BPI and LBP (BPIFD1 and BPIFD2) are highly conserved both in sequence and structure, whereas other BPI-fold proteins show moderate sequence divergence (Table 2) but retain the core boomerang-shaped architecture characteristic of the family (Figure 2). Genomic analyses indicate that this family expanded through gene duplication within the human chromosome 20q11.21 region, generating several BPI-like proteins that are expressed in the olfactory epithelium and Bowman’s glands, including BPIFB3 and BPIFB4 [86].

BPIFB4 has been consistently detected in proteomic analyses of olfactory mucus and is expressed in Bowman’s glands, the primary secretory source of the mucus, as well as in mononuclear cells, suggesting potential roles in chemical defense and mucosal homeostasis [86]. BPIFB3, another member of the same subgroup, has been more extensively characterized functionally. It has been shown to regulate a noncanonical autophagy pathway and suppress coxsackievirus B replication, indicating that BPI-fold proteins can contribute not only to lipid binding but also to broad cellular defense mechanisms [87]. These findings indicate that BPIFB3 and BPIFB4 are likely to play important roles in innate defense and mucosal surfactant activity [52,88]. In addition, BPIFA1 (also known as SPLUNC1) regulates epithelial ion transport through the pH-dependent inhibition of ENaC, thereby contributing to airway surface liquid homeostasis and protecting against bacterial colonization. Reduced SPLUNC1 expression has been linked to impaired mucosal defense, highlighting its importance as a functional component of the upper airway secretome. Beyond their mucosal protective roles, several BPIF family members have also been implicated in upper airway cancers. Notably, decreased expression of BPIFA1 and BPIFB1 has been observed in nasopharyngeal carcinoma (NPC) biopsy samples [89]. Furthermore, genetic and functional studies have demonstrated that *BPIFA1* variants are associated with NPC susceptibility [90] and that loss of BPIFA1 expression correlates with poorer prognosis and reduced retinoic acid–induced growth inhibition and differentiation in NPC cells [91], suggesting that this protein family may play tumor-suppressive and mucosal-protective roles.

### 3.4. Proteins Supporting Stress Resilience and Barrier Integrity

The resilience of the olfactory mucosa is maintained by a diverse repertoire of protective proteins, especially in the context of stress or inflammatory conditions. Among these protective factors, heat shock proteins (HSPs) function as key molecular chaperones that maintain protein homeostasis when the olfactory epithelium is exposed to environmental toxins, high concentrations of odorants, or oxidative stress. Although classically regarded as intracellular proteins, HSPs can be released via vesicles (exosomes/oncosomes), where they have been shown to participate in immune regulation [92]. Consistent with that finding, proteomic analyses of human olfactory mucus have identified multiple HSP family members within the mucus environment [52,53,79]. Moreover, HSP70 is expressed in olfactory receptor neurons, sustentacular cells, and Bowman’s glands, where it facilitates the proper folding and trafficking of olfactory receptor proteins and enhances neuronal resilience to stress [64]. Similarly, HSP25/27 are strongly induced in sustentacular cells that have been exposed to high concentrations of odorants, where they support detoxification enzyme systems and help preserve epithelial barrier integrity [65]. In addition to HSPs, protective proteins such as SLPI (secretory leukocyte protease inhibitor) and MUC5B (mucin 5B) help to maintain mucosal barrier integrity. Furthermore, perireceptor metabolic enzymes constitute a critical defense system against reactive compounds. They include UGT2A1/2 (UDP-glucuronosyltransferases), ALDHs (aldehyde dehydrogenases), AKRs (aldo-keto reductases), and GSTP1 (glutathione S-transferase P1) and metabolize reactive aldehydes to protect the epithelium and stabilize odorant signaling [62].

## 4. Extracellular Matrix (ECM)–Protein Networks in the Human Olfactory Cleft

### 4.1. Roles of Soluble and Structural ECM Components in Olfactory Sensation

**Figure 3:**
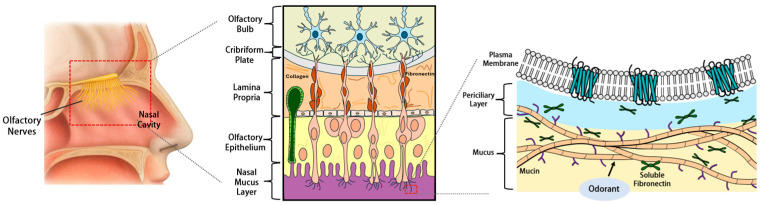
Protein networks spanning the mucus-epithelium-extracellular matrix interface in the human olfactory system. Schematic representation illustrating the multi-compartment organization of the olfactory mucosa. (**Left**) Anatomical overview showing the nasal cavity and olfactory region. (**Middle**) Layered architecture depicting the nasal mucus layer (purple, bottom) containing soluble proteins, the olfactory epithelium (yellow, middle) comprising sensory neurons and supporting cells, and the lamina propria (blue, top) housing Bowman’s glands and connective tissue. (**Right**) Molecular detail of the mucus-epithelium-ECM interface. Extracellular matrix components exhibit dual localization: fibronectin exists as soluble plasma fibronectin in the olfactory mucus (apical region, near plasma membrane) where it directly interacts with odorants and modulates receptor sensitivity, and as structural cellular fibronectin in the lamina propria ECM (basal region) where it forms fibrillar networks providing mechanical support. Odorant molecules (blue spheres) are confined to the mucus layer where they interact with soluble carrier proteins and ECM-derived molecules. Structural collagen fibers (orange) are predominantly localized to the lamina propria beneath the basement membrane, providing tissue scaffolding and regulating epithelial homeostasis. This organization reflects recent findings that ECM proteins participate in perireceptor events through their soluble forms in addition to their traditional structural roles, with fibronectin and other ECM components consistently detected in olfactory mucus by proteomic analyses.

Within the human olfactory cleft, the ECM functions beyond structural support to actively regulate odorant detection, tissue homeostasis, and disease progression. Recent evidence demonstrates that ECM components cooperate with OBP-related proteins to form a dynamic network that influences both physiological olfactory function and pathological remodeling. Fibronectin has emerged as a critical perireceptor modulator in human olfaction. Although fibronectin is classically known as a structural ECM protein in the lamina propria, a soluble form derived in part from Bowman’s glands is consistently detected in human olfactory epithelial mucus. Fibronectin purified from human olfactory epithelial mucus has been shown to enhance the sensitivity of heterologously expressed olfactory receptors and to partially restore electroolfactogram responses after mucus removal [78]. Importantly, fibronectin levels are reduced in idiopathic olfactory dysfunction, which establishes a direct link between ECM integrity and smell disorders [78]. This finding is consistent with the broader paradigm that understands ECM as an active driver of tissue behavior, with composition and mechanical properties that are tightly coupled to cellular signaling pathways [93]. We depicted ECM components in the human olfactory system as shown in Figure 3.

### 4.2. ECM Remodeling Circuits

The fibronectin–collagen interface provides particularly important instructive cues. Continuous fibronectin–collagen binding is required to sustain proper cell proliferation and microtissue morphology, demonstrating how matrix–matrix interactions contribute to shaping the perireceptor niche [94]. Clinical studies in asthmatic airways have shown that reduced fibronectin production impairs epithelial repair dynamics, with FN–α5β1 integrin engagement being essential for effective wound closure. These findings suggest that fibronectin deficiency could disrupt not only tissue repair but also the distribution of secreted carrier proteins within olfactory mucus [95,96]. Among the lipocalins, LCN2 (NGAL) exemplifies how these proteins can actively influence ECM remodeling. LCN2 binds matrix metalloproteinase-9 (MMP-9), preventing its autolytic degradation and stabilizing its proteolytic activity against ECM substrates such as collagen and fibronectin. This LCN2–MMP-9 complex has been documented in the urine of human cancer patients and synovial fluid from osteoarthritis patients, indicating its broad pathophysiological relevance [97,98]. In upper aerodigestive tract carcinomas, differential expression of LCN2, MMP-9, and their circulating complex supports their potential utility as biomarkers in head-and-neck-adjacent tissues [99]. The mechanism becomes even more intricate with the involvement of LOXL2 (lysyl oxidase-like 2). The extracellular LCN2–LOXL2–MMP-9 triad has been shown to accelerate fibronectin and Matrigel degradation and simultaneously activate FAK–AKT–GSK3β signaling, demonstrating coordinated matrix degradation coupled with mechanotransduction [100]. Post-transcriptional regulation has also emerged as an important mechanism in ECM remodeling and mucosal defense. One such mechanism is mediated by miR-761, which downregulates LCN2/Twist1 expression, thereby attenuating the epithelial–mesenchymal transition (EMT) and matrix reprogramming in chronic rhinosinusitis models. These findings highlight the contribution of microRNA-mediated pathways to the fine-tuning of olfactory mucosal remodeling and suggest that similar mechanisms could influence OBP- and lipocalin-associated networks in the olfactory niche [101].

### 4.3. Direct Interactions Between Carrier Proteins and ECM

Direct carrier–matrix interactions are not limited to lipocalins. Apolipoprotein A-I, which is consistently detected in olfactory cleft mucus, binds fibronectin and collagen I through saturable interactions and associates with ECM fibers in tissue sections. Apolipoproteins are present in both mucus-soluble and matrix-associated forms. Soluble apolipoproteins in olfactory cleft mucus can directly bind and transport hydrophobic odorants to receptors, while matrix-associated forms, such as APOA1 binding to extracellular matrix proteins [102], may primarily function in lipid homeostasis and metabolite clearance within the lamina propria rather than direct odorant interaction. These distinct compartmental roles suggest coordinated lipid management across the mucus-epithelium-matrix interface. In addition, lipocalin-derived peptides have been shown to directly stimulate ECM production by increasing collagen, fibronectin, and tenascin synthesis in fibroblasts, demonstrating the presence of bidirectional crosstalk between lipocalins and the ECM [103]. Within the BPI-fold family, BPIFB4 provides compelling evidence of tissue-protective functions. The longevity-associated variant (*LAV-BPIFB4*) has been shown to support cardiac function and vascularization in age-related cardiomyopathy and modulate myocardial fibrosis. Related interventions with longevity-associated proteins have also been reported to improve function in murine models of heart failure [104,105,106]. Although those findings have been derived primarily from cardiovascular studies, they establish the broader principle that BPI-fold proteins can reprogram tissue microenvironments, suggesting the potential for similar roles within the olfactory system.

## 5. Pathological Alterations in Olfactory Cleft Proteins

### 5.1. Smell Loss Disorders (Idiopathic and Age-Related)

In insects, the loss of OBPs has been shown to abolish the recognition of specific pheromones and the associated behaviors. For instance, *Drosophila* mutants lacking OBP76a (LUSH) are unable to detect the male pheromone cVA and consequently exhibit impaired pheromone-mediated attraction behaviors [39,107]. Similarly, knockout of PBP1 in the silk moth *Bombyx mori* results in a reduced response to the sex pheromone bombykol [108]. In contrast, classical OBPs or OBP-like proteins do not appear to act as direct mediators of anosmia in humans. Instead, several proteins and ECM components, many of which are closely associated with aging and neurodegeneration, have emerged as key factors in preserving olfactory sensitivity and epithelial integrity. Notably, fibronectin levels are reduced in patients with idiopathic olfactory disorders, which provides a direct link between ECM integrity and olfactory dysfunction [78].

LCN15, which is secreted from Bowman’s glands into the olfactory mucus, has been implicated in maintaining the integrity of olfactory sensory neurons. Ijichi et al. (2022) reported that LCN15 is strongly expressed in young adults (20–40 years) but is significantly reduced in the nasal mucus of elderly individuals (≥60 years), in parallel with degeneration of olfactory sensory neurons and associated olfactory impairment [54]. In addition, perireceptor metabolic enzymes such as UGT2A1/2, ALDHs, and AKRs contribute to odorant clearance and detoxification, thereby stabilizing olfactory signaling and protecting the epithelium. Notably, genetic variants in the *UGT2A1/UGT2A2* locus have been associated with COVID-19–related anosmia, underscoring the clinical relevance of this metabolic pathway in human olfactory dysfunction [109]. Together, these findings highlight that both lipocalins and metabolic enzymes are critical determinants of olfactory resilience. Age-related changes in HSP expression further illustrate the complex interplay between aging and neurodegeneration. In human olfactory receptor neurons, HSP70 expression declines with age, and that reduction is more pronounced in patients with Alzheimer’s disease than in those without it, suggesting that impaired HSP70 expression contributes to diminished resilience and heightened neuronal vulnerability [110]. In contrast, studies in the olfactory bulbs of aging mice have reported increased basal expression of HSP27 and HSP70, indicating compensatory activation of stress-response pathways [111]. Furthermore, in neurodegenerative diseases such as Alzheimer’s and Parkinson’s diseases, HSP70 and HSP90 are typically upregulated in response to toxic protein accumulation, thereby supporting proteostasis and contributing to neuronal defense [66,112].

### 5.2. Nasal Inflammatory Diseases

Chronic rhinosinusitis and allergic rhinitis are among the most common chronic inflammatory disorders of the upper airway and are closely associated with altered protein expression in the olfactory epithelium [59]. Olfactory dysfunction is a hallmark symptom of chronic rhinosinusitis, affecting 60–80% of patients, and is directly linked to a substantial reduction in quality of life [113]. In physiological conditions, a variety of mucosal proteins and defense factors regulate oxidative stress and maintain epithelial barrier integrity. In chronic rhinosinusitis and allergic rhinitis, however, dysregulated expression of those proteins compromises host defense and contributes to olfactory dysfunction [60,61,114]. Oxidative stress plays a central role in disease persistence and has been linked to mucin overproduction, the induction of stress-response proteins, and alterations in lipid-binding proteins. Notably, APOA1, APOA2, APOE, and clusterin (ApoJ) are selectively elevated in allergic rhinitis, where they might contribute to lipid transport and have anti-inflammatory functions [115].

In patients with chronic rhinosinusitis with nasal polyps (CRSwNP), LCN2 is markedly upregulated in association with the IL-17/IL-8 inflammatory axis. Functionally, LCN2 binds to MMP-9, preventing its degradation and thereby prolonging its enzymatic activity. Sustained MMP-9 activity has been shown to promote LPS-induced MUC5AC expression, which contributes to mucus hypersecretion, ECM degradation, and mucosal thickening [63]. Given the pathogenic role of LCN2 in CRSwNP, its regulation is likely to represent a potential therapeutic strategy. For example, miR-761 has been reported to downregulate both LCN2 and Twist1 expression, thereby suppressing the EMT and attenuating mucosal remodeling [101]. Notably, the expression of several innate immune proteins, including SPLUNC1 (BPIFA1), LPLUNC2 (BPIL-1), BPIFB1, BPIFB2, lysozyme, and SLPI, is reduced in CRSwNP. This downregulation weakens antibacterial defenses, facilitates persistent bacterial colonization, and contributes to the chronic inflammatory milieu characteristic of the disease [57,58].

### 5.3. Infection and Environmental Stress Defense

Several proteins within the olfactory mucus serve critical functions in innate immunity against external pathogens. Among them, members of the lipocalin and BPI-fold protein families contribute to host defense through both extracellular ligand binding and intracellular regulatory pathways. For instance, LCN1 enhances antimicrobial defense by sequestering bacterial and fungal siderophores, thereby restricting microbial access to iron [75]. Similarly, LCN2 (NGAL) limits bacterial growth by binding siderophores [55], and its critical role has been validated in infection models in which LCN2-deficient mice displayed markedly increased bacterial burden and mortality during *Klebsiella pneumoniae* pneumonia [77]. Beyond antibacterial defense, BPI-fold proteins contribute to antiviral immunity. For example, BPIFB3 restricts coxsackievirus B replication by suppressing a noncanonical autophagy pathway, and loss of BPIFB3 results in excessive autophagy activation and enhanced viral replication. Collectively, these findings indicate that BPI-fold proteins extend their functions beyond lipid binding to encompass broad intracellular defense mechanisms [87]. In addition to pathogens, the olfactory epithelium must also withstand a variety of environmental stressors. Exposure to high concentrations of odorants, nonspecific toxins, and oxidative stress triggers the induction of protective stress proteins. For example, HSP25 is strongly upregulated in sustentacular cells following odorant overexposure, and it supports detoxification enzyme systems and helps preserve epithelial barrier integrity [65]. Likewise, odorant-induced HSP70 expression is observed in sustentacular cells and Bowman’s glands but not in olfactory receptor neurons, highlighting the importance of non-neuronal cells in protecting sensory neurons from environmental stress [64]. Together, these findings highlight the role of stress proteins as key mediators of epithelial resilience in the olfactory system. Table 3 presents a comprehensive summary of olfactory system disorders resulting from specific protein and gene changes, as discussed in Section 5.

## 6. Controversies and Knowledge Gaps

### 6.1. Controversy over the Essentiality of OBPs in Humans

In insects, the loss of specific OBPs directly abolishes recognition of the corresponding pheromones and produces behavioral deficits, providing strong evidence for their indispensability. In humans, on the other hand, the presence and essentiality of the classical OBPs (OBP2A/2B) have not been consistently demonstrated. Although OBP2A/2B transcripts were reported in early studies, their proteins are rarely detected in human olfactory mucus, and their physiological contribution to odorant detection remains uncertain.

Nevertheless, although differences in detection sensitivity and sampling procedures may exist across studies, proteomic analyses have repeatedly failed to detect OBP2A and OBP2B proteins in human olfactory mucus, leading to a prevailing view that the two classical OBPs are essentially dispensable for human olfaction. Human olfactory mucus sampling presents inherent methodological challenges compared to rodent models, including variability in sampling location, mucus dilution, and protein recovery efficiency. However, the absence of classical OBPs likely reflects genuine biological scarcity rather than purely technical limitations, as multiple independent studies using high-sensitivity mass spectrometry have consistently detected other low-abundance proteins such as LCN15 [54] and BPI-fold proteins [52] in the same samples. Furthermore, transcriptomic analyses demonstrate substantially lower expression of OBP2A and OBP2B compared to LCN1 and LCN15 [116], supporting this interpretation.

Furthermore, although OBP-like proteins are detectable in olfactory cleft mucus, post-translational modifications such as glycosylation and phosphorylation, as described for porcine OBPs, can obscure isoform-specific detection and complicate functional interpretation in humans. These limitations impose fundamental challenges for defining the precise role of human OBPs. Thus, whether OBP-like proteins are essential for human olfaction remains unresolved. Notably, LCN15 has been shown to enhance the responses of specific receptor–odorant pairs (e.g., OR51E1–isovaleric acid), suggesting that some OBP-like proteins function not merely as general carriers but as receptor-specific co-factors. This finding supports the emerging view that OBP-like proteins act as functional modulators within the human olfactory system rather than passive odorant carriers. However, without systematic mapping of receptor–protein interactions, it remains unclear which receptors OBP-like proteins modulate in the human olfactory system.

### 6.2. Crosstalk Between the Olfactory Mucus Proteome and Neurodegeneration

Apolipoproteins such as ApoD are abundantly expressed in the central nervous system and are implicated in neurodegenerative diseases such as Alzheimer’s and Parkinson’s diseases through their regulation of oxidative stress and lipid metabolism. Interestingly, these proteins have also been detected in human olfactory cleft mucus, suggesting a potential link to the olfaction–brain axis. Reduced apolipoprotein expression in neurodegenerative diseases parallels early olfactory dysfunction, suggesting a potential olfactory–neurodegeneration connection. Moreover, interactions between olfactory sensory neurons (OSNs) and gland-secreted proteins such as LCN15 suggest a functional coupling between secretory and neuronal components, indicating that the olfactory mucus proteome actively contributes to neuronal maintenance and signal stability beyond its traditional role in odorant transport. Similarly, ECM components, particularly fibronectin and collagen networks, enhance odorant receptor sensitivity and maintain the structural integrity of the OSN–mucus interface, thereby supporting neuronal preservation and sensory sensitivity. Thus, ECM degradation or loss is likely to represent a pathway that exacerbates olfactory decline in neurodegenerative diseases by promoting OSN damage.

Although direct evidence linking neurodegenerative disorders to specific alterations in the olfactory mucus proteome remains limited, several studies have identified molecular correlations suggesting potential mechanistic connections. Notably, HSP70 levels decline with aging and in Alzheimer’s disease, while LCN15 expression is reduced in both aged individuals and those with idiopathic olfactory loss. These proteins are increasingly regarded as potential early biomarkers that reflect the structural and functional integrity of the olfactory and neuronal systems. Beyond protein-based markers, alterations in ECM components provide an additional dimension for understanding how molecular and structural remodeling within the olfactory mucus contributes to sensory decline and neurodegenerative progression. This gap underscores the potential of olfactory proteomics, beyond classical chemosensory biology, to provide a framework for exploring the ECM–protein–neural function axis and its contribution to neurodegenerative disease.

### 6.3. Conclusion and Future Perspectives

Human OBPs and OBP-like proteins provide a unique interface for the chemical environment, mucosal defense, and neuronal function. Although insect studies firmly establish OBPs as indispensable for pheromone detection, human OBPs display a more complex and context-dependent role. Proteomic analyses have highlighted LCN1, LCN2, LCN15, and ApoD as consistent components of the olfactory mucus, where they support odorant transport, oxidative stress regulation, and immune defense. In addition, ECM–protein interactions, exemplified by fibronectin, collagen, and lipocalin–matrix complexes, are emerging as critical determinants of olfactory sensitivity and resilience. Pathological alterations in those proteins have been linked to diverse conditions, including idiopathic smell loss, aging, chronic rhinosinusitis, and neurodegenerative diseases such as Alzheimer’s and Parkinson’s diseases. However, major gaps remain in understanding the indispensability of OBPs in humans, their receptor-specific interactions, and the direct causal relationship between olfactory proteome alterations and central neurodegenerative processes.

Future research in three major directions is needed. First, molecular mapping of OBP–receptor interactions, supported by single-cell transcriptomics and high-resolution proteomics, is essential to clarify their contribution to odorant specificity, especially given the potential impact of post-translational modifications on binding affinity and receptor selectivity. Second, combining structural biology with functional assays will help elucidate how OBP–receptor interactions, as well as protein–ECM associations, shape OBP function. Third, clinical translation requires longitudinal studies to evaluate OBPs and their related proteins as biomarkers for the early detection of olfactory dysfunction and neurodegenerative disease. By bridging chemosensory biology to mucosal immunology and neurodegeneration research, OBPs and OBP-like proteins could ultimately be positioned as key molecular mediators at the intersection of olfactory perception, host defense, and brain health.

## Figures and Tables

**Table 1:** Proteomic Landscape of the Human Olfactory Cleft.

Protein Category ^a^	Representative Proteins (Gene Symbols) ^b^	Primary Functions in Olfactory Mucus ^c^	Detection and Characteristics ^d^	Primary Detection Method ^e^	Key References ^f^
Classical OBP	OBP2A, OBP2B	Binding and transport of hydrophobic odorants	Variably reported in OC mucus depending on study; some ELISA-positive reports, often undetected in proteomics	Genomic identification; ELISA	[47,50,51]
Lipocalin (OBP-like)	LCN15	Odorant binding	Highly expressed in OC mucus, secreted from Bowman’s glands, linked to age, inflammation, and olfactory dysfunction	LC-MS/MS proteomics of olfactory mucus	[54]
	LCN2	Antibacterial/Defense	Linked to inflammatory OC mucus	LC-MS/MS proteomics	[55]
	LCN1	Chemical defense	Reported in some proteomic studies	LC-MS/MS proteomics	[56]
PLUNC/BPI-fold	BPIFB4, BPIFA1(SPLUNC1), BPIFB2	Innate defense, Surface tension regulation, Ion balance	Repeatedly detected in OC/non-OC mucus proteomes (BPIFB4 especially abundant)	LC-MS/MS proteomics of nasal/olfactory mucus	[57,58]
Apolipoprotein	APOA1/2/4, APOB100, APOE, CLU	Lipid transport, Protein quality control, Anti-inflammatory defense	Repeatedly reported across cohorts; APOA1/2 strongly link to AR	LC-MS/MS proteomics	[59,60,61]
Peri-receptor Enzymes (ODE)	UGT2A1/2, ALDHs, GSTP1, CES, CYPs	Odorant metabolism, Detoxification	Repeatedly identified in AR/OC/CRS mucus proteomes; included in multiple proteomic datasets	LC-MS/MS proteomics	[62]
Barrier/Defense proteins	SLPI, MUC5B	Mucosal barrier maintenance, Antimicrobial activity	Detected in AR/CRS OC mucus; interpreted as barrier weakness	LC-MS/MS proteomics	[58,63]
Chaperones/Stress proteins	HSP70, HSP27/25	Protein quality control, Stress response	Altered expression under inflammatory and stress conditions	Immunocytochemistry; Immunohistochemistry	[64,65,66]

Proteins consistently detected in human olfactory cleft mucus, organized by functional category. This table presents a functionally curated subset emphasizing proteins with established relevance to perireceptor events. ^a^ Protein Category: Functional classification based on primary biochemical role and structural family membership. ^b^ Representative Proteins (Gene Symbols): Major protein members detected in proteomic studies of human olfactory cleft mucus. Gene symbols follow HGNC nomenclature. ^c^ Primary Functions in Olfactory Mucus: Experimentally demonstrated or strongly proposed roles in perireceptor events, including odorant binding/transport, mucosal defense, metabolic processing, and tissue homeostasis. ^d^ Detection and Characteristics: Summary of proteomic detection consistency across studies, expression patterns, post-translational modifications, and notable biochemical properties relevant to olfactory physiology. ^e^ Primary Detection Method: Summary of predominant experimental approach used across the cited studies, with full methodological details provided in the corresponding references. ^f^ Key References: Selected primary literature demonstrating protein identification, characterization, or functional studies in olfactory or nasal mucus.

**Table 2:** Sequence Identity and Similarity Analysis of Human BPI-fold Superfamily.

Subfamily	Full Protein Name	Alias	Identity	Similarity
BPIFD1	Bactericidal permeability-increasing protein precursor	BPI	100	100
BPIFD2	Lipopolysaccharide-binding protein precursor	LBP	45.2	65.2
BPIFE	Phospholipid transfer protein	PLTP, HDLCQ9	25.8	45.4
BPIFF	Cholesteryl ester transfer protein	CETP, HDLCQ10	23	40.9
BPIFA1	BPI fold–containing family A member 1	SPLUNC1/PLUNC	12.9	21
BPIFA2	BPI fold–containing family A member 2	LPLUNC2, BPIL1, C20orf184, RYSR,	9.6	18
BPIFA3	BPI fold–containing family A member 3	SPLUNC3, C20orf71	9.9	16.2
BPIFB1	BPI fold–containing family B member 1	LPLUNC1, C20orf114	19.2	36.3
BPIFB2	BPI fold–containing family B member 2	LPLUNC2, BPIL1, C20orf184, RYSR	21.9	43
BPIFB3	BPI fold–containing family B member 3	LPLUNC3, C20orf185, RYA3	20	38.1
BPIFB4	BPI fold–containing family B member 4	LPLUNC4, C20orf186, RY2G5	14.7	28.4
BPIFB6	BPI fold–containing family B member 6	BPIL3, LPLUNC6	22.1	39.2
BPIFC	BPI fold–containing family C protein	BPIL2	26.6	46.4

Sequence analysis reveals evolutionary divergence within the BPI-fold superfamily, with identity ranging from 9.6% to 45.2% despite conservation of the characteristic boomerang-shaped fold architecture. BPIFA members (single domain) show lowest identity (9.6–12.9%), while BPIFB members (two domains) show moderate conservation (14.7–22.1%). LBP exhibits the highest similarity to BPI (45.2% identity), consistent with their shared roles in lipopolysaccharide recognition.

**Table 3:** Disease- and Condition-Associated Protein/Gene Changes in the Olfactory System.

Disease/Condition ^a^	Affected Proteins or Genes ^b^	Expression Change (↑/↓) ^c^	Tissue/Sample Source ^d^	Key References ^e^
Aging	LCN15	↓ (aged; idiopathic loss tendency	Human, nasal mucosa	[54]
	HSP70	↓ in ORNs (not in sustentacular cells or Bowman’s glands)	Human, olfactory mucosa (immunocytochemistry)	[110]
Alzheimer’s disease	HSP70	↓ in ORNs (not in sustentacular cells or Bowman’s glands)	Human, Human, olfactory mucosa (immunocytochemistry)	[110]
Viral infection	*UGT2A1/2*	SNP (rs7688383 T allele) ↑ risk of COVID-19-related smell/taste loss	Human, genome-wide association study	[109]
	BPIFB3	↓ → CVB replication ↑; ↑ → replication ↓	Human cell lines (HBMEC, U2OS, HeLa, 786-O; Coxsackievirus B infection model	[87]
Bacterial infection	LCN2	Infection → LCN2 ↑ → bacterial growth ↓	In vitro (*E. coli* growth assay); mouse nasal infection model (*K. pneumoniae* KPPR1)	[55]
Allergic rhinitis	APOA1, APOA2, APOE, APOJ	↑	Human, nasal mucosa and nasal fluid	[101]
	HSP70	↑	Human, nasal mucosa (mRNA)	[114]
CRS/CRSwNP	MUC3, MUC6	↓	Human, nasal mucosa	[60]
	MUC1, MUC2, MUC4, MUC5AC, MUC5B, MUC8	↑	Human, nasal mucosa	[60]
	Fibronectin	↓ (also in Idiopathic olfactory disorder)	Human, olfactory mucosa	[78]
	LCN2	↑ in CRSwNP (human); ↓ by miR-761 (mouse)	Human, CRSwNP tissue; Mouse, nasal mucosa	[63,101]
	BPIFA1/SPLUNC1, BPIFB1, BPIFB2/LPLUNC2, BPIL-1, SLPI, CLU, LTF, LYZ	↓	Human, CRSwNP tissue	[57,58]
Environmental stress	HSP25	↑ (odorant-induced)	Mouse, olfacoty mucosa	[65]
	HSP70	↑ (odorant-induced)	Rat, olfacoty mucosa	[64]
Axillary osmidrosis	ApoD	↑	Human, apocrine gland(mRNA)	[80,81]

Proteins consistently detected in human olfactory cleft mucus, organized by functional category. This table presents a functionally curated subset emphasizing proteins with established relevance to perireceptor events. ^a^ Disease/Condition: Clinical or experimental condition affecting olfactory function, including aging, inflammatory disorders, neurodegenerative diseases, and genetic variations. ^b^ Affected Proteins or Genes: Specific proteins or genes showing altered expression, activity, or localization. Protein names refer to mature secreted forms; gene symbols (italics) refer to transcriptional changes. ^c^ Expression Change (↑/↓): Direction and magnitude of change relative to healthy controls. ↑ indicates upregulation/increased expression; ↓ indicates downregulation/decreased expression. Includes mRNA, protein levels, or functional activity where specified. ^d^ Tissue/Sample Source: Biological material analyzed, including human clinical samples (nasal mucosa, olfactory cleft mucus), animal models, or cell culture systems. Specifies whether data derive from in vivo, ex vivo, or in vitro studies. ^e^ Key References: Primary studies reporting the association, including proteomic, transcriptomic, or immunohistochemical analyses.

## Data Availability

No new data were created or analyzed in this study.

## Abbreviations

The following abbreviations are used in this manuscript:

OBP	Odorant-binding protein
OBP2A/2B	Human odorant-binding proteins 2A/2B
LCN	Lipocalin
LCN1	Lipocalin-1
LCN2	Lipocalin-2 (NGAL)
LCN15	Lipocalin-15
ApoD	Apolipoprotein D
ApoA1/A2/A4	Apolipoproteins A-I/A-II/A-IV
ApoE	Apolipoprotein E
CLU (ApoJ)	Clusterin (Apolipoprotein J)
BPI	Bactericidal/permeability-increasing protein
LBP	Lipopolysaccharide-binding protein
PLUNC	Palate, lung, and nasal epithelium clone protein family
BPIFA1 (SPLUNC1)	BPI fold-containing family A member 1
BPIFB2 (LPLUNC2)	BPI fold-containing family B member 2
BPIFB3/BPIFB4	BPI fold-containing family B members 3/4
ECM	Extracellular matrix
OSN	Olfactory sensory neuron
OR	Olfactory receptor
GPCR	G-protein–coupled receptor
EOG	Electro-olfactogram
MMP-9	Matrix metalloproteinase-9
MUPs	Major urinary proteins
LOXL2	Lysyl oxidase-like 2
FAK	Focal adhesion kinase
AKT	Protein kinase B
GSK3β	Glycogen synthase kinase-3 beta
EMT	Epithelial–mesenchymal transition
UGT2A1/2	UDP-glucuronosyltransferases 2A1/2
ALDH	Aldehyde dehydrogenase
AKR	Aldo-keto reductase
GSTP1	Glutathione S-transferase P1
CES	Carboxylesterase
CYP	Cytochrome P450
HSP	Heat shock protein
CRS	Chronic rhinosinusitis
CRSwNP	Chronic rhinosinusitis with nasal polyps
AR	Allergic rhinitis
ENaC	Epithelial sodium channel
cVA	cis-Vaccenyl acetate
PBP1	Pheromone-binding protein 1
SNP	Single-nucleotide polymorphism
OC	Olfactory cleft
